# Implementation of LGBTQ+ affirming care policies in the Veterans Health Administration: preliminary findings on barriers and facilitators in the southern United States

**DOI:** 10.3389/fpubh.2023.1251565

**Published:** 2024-01-30

**Authors:** Rajinder Sonia Singh, Sara J. Landes, Cathleen E. Willging, Traci H. Abraham, Pamela McFrederick, Michael R. Kauth, Jillian C. Shipherd, JoAnn E. Kirchner

**Affiliations:** ^1^Center for Mental Healthcare and Outcomes Research, Central Arkansas Veterans Healthcare System, North Little Rock, AR, United States; ^2^Department of Psychiatry, University of Arkansas for Medical Sciences, Little Rock, AR, United States; ^3^VA Behavioral Health Quality Enhancement Research Initiative, Central Arkansas Veterans Healthcare System, Little Rock, AR, United States; ^4^South Central Mental Illness Research, Education and Clinical Center, Houston, TX, United States; ^5^Behavioral Health Research Center of the Southwest, Pacific Institute for Research and Evaluation, Albuquerque, NM, United States; ^6^Clinical Outcome Assessments, Clinical Outcomes Solutions, Chicago, IL, United States; ^7^South Central VA Health Care Network, Ridgeland, MS, United States; ^8^LGBTQ+ Health Program, Department of Veterans Affairs, Washington, DC, United States; ^9^Baylor College of Medicine, Houston, TX, United States; ^10^Boston University Chobanian & Avedisian School of Medicine, Boston, MA, United States; ^11^National Center for PTSD, VA Boston Healthcare System, Boston, MA, United States

**Keywords:** LGBTQ+, policy implementation, inequity, veterans, implementation

## Abstract

**Background:**

In the United States Department of Veterans Affairs (VA), veterans who are lesbian, gay, bisexual, transgender, queer, and similar gender and sexual minoritized people (LGBTQ+) experience health disparities compared to cisgender, heterosexual veterans. VA’s LGBTQ+ Health Program created two healthcare policies on providing LGBTQ+ affirming care (healthcare that is inclusive, validating, and understanding of the LGBTQ+ population). The current project examines providers’ barriers and facilitators to providing LGBTQ+ affirming care and LGBTQ+ veterans’ barriers and facilitators to receiving LGBTQ+ affirming care.

**Methods:**

Data collection and analysis were informed by the Consolidated Framework for Implementation Research, which was adapted to include three health equity domains. Data collection involved telephone interviews conducted with 11 VA providers and 12 LGBTQ+ veterans at one rural and one urban VA medical center, and one rural VA community clinic. Qualitative data were rapidly analyzed using template analysis, a data reduction technique.

**Results:**

Providers described limited education, limited time, lack of experience with the population, and a lack of awareness of resources as barriers. Providers discussed comfort with consulting trusted peers, interest in learning more about providing LGBTQ+ affirming care, and openness and acceptance of the LGBTQ+ community as facilitators. LGBTQ+ veterans described a lack of provider awareness of their needs, concerns related to safety and discrimination, and structural discrimination as barriers. LGBTQ+ veterans described positive relationships with providers, knowledge of their own healthcare needs, and ability to advocate for their healthcare needs as facilitators. Although VA’s LGBTQ+ affirming care policies are in place, providers and veterans noted a lack of awareness regarding specific healthcare processes.

**Conclusion:**

Allowing more time and capacity for education and engaging LGBTQ+ veterans in determining how to improve their healthcare may be the path forward to increase adherence to LGBTQ+ affirming care policies. Engaging patients, especially those from marginalized backgrounds, in strategies focused on the uptake of policy may be a path to improve policy implementation. It is possible that creating truly collaborative structures in which patients, staff, providers, leadership, and policymakers can work together towards policy implementation may be a useful strategy. In turn, improved policy implementation would result in increased physical and mental health for LGBTQ+ veterans.

## 1 Introduction

Lesbian, gay, bisexual, transgender, queer, and similar gender and sexual minoritized (LGBTQ+) people experience health disparities disproportionately compared to cisgender, heterosexual people (1, 2). Similarly, LGBTQ+ veterans experience greater health disparities than cisgender, heterosexual veterans (3, 4). These health disparities include a greater prevalence of certain mental health problems, including alcohol and substance use disorders, anxiety, depression, posttraumatic stress disorder (PTSD), and higher rates of suicidal ideation and attempts (3, 5–9). LGBTQ+ veterans also experience disparities in social stressors, including decreased emotional and social support and higher rates of homelessness and military sexual trauma (4, 5).

Having other intersecting marginalized identities, in addition to being LGBTQ+, can place veterans at heightened risk for health disparities due to the effects of discrimination. Additional marginalized identities may be shaped by race, ethnicity, socioeconomic status, and place of residence to name a few. Previous research on health disparities for Black and Hispanic veterans indicates that they experience worse health and greater combat exposure compared to white Veterans (10). Rural veterans are more likely to experience health disparities than their urban counterparts as a result of less accessible and available healthcare and public health services, lower socioeconomic statuses, and lower health literacy (11–13). Overall, research on LGBTQ+ veterans with intersecting marginalized identities is sparse. The limited literature indicates that Black transgender veterans are more likely to be socially disadvantaged and experience more mental health and medical conditions compared to white transgender veterans (14). Veterans who are gay men in rural settings report greater depressive and anxiety symptoms and greater tobacco use than their suburban/urban counterparts (15).

Minority stress theory helps explain the causes and consequences of health disparities (16–18). This theory posits that minority group members, such as LGBTQ+ people, experience distinct and chronic stressors due to the societal response to their social identities, including identities based on sexual orientation or gender (16–18). The excessive and cumulative toll of structural factors, such as social stigma, discrimination, and prejudice, experienced by LGBTQ+ people can adversely impact mental and physical health and overall well-being. These same factors can undermine access to and utilization of healthcare by LGBTQ+ people, contributing to unmet needs (19). Historically, healthcare systems in the United States pathologized and discriminated against LGBTQ+ people by applying stigmatizing psychiatric diagnoses and refusing to care for patients with HIV/AIDS (19). Providers may hold negative attitudes toward and lack knowledge about LGBTQ+ people and often report feeling ill-prepared to provide high-quality care to this population (19, 20).

Provider perceptions and understanding of LGBTQ+ people and their healthcare needs may vary based on the setting in which clinical care occurs (e.g., rural facility). Research examining Veterans Health Administration (VHA) providers’ viewpoints on LGBTQ+ veterans is limited. However, civilian examinations of provider perceptions and understanding of LGBTQ+ people and their healthcare needs indicate variations in rural provider understanding of LGBTQ+ patient needs (21, 22). For example, rural, civilian healthcare providers reported no difference in how they approach working with LGBTQ+ and non-LGBTQ+ patients (22). Studies of rural providers also illustrate a lack of knowledge and preparation related to addressing LGBTQ+ patient needs, including limited formal education and minimal training upon entering their respective professions (21, 22). Rural providers report that they are less likely to work with openly LGBTQ+ people in their practice (23). LGBTQ+ people in rural-dwelling places are more likely to experience disparities. Because most research on LGBTQ+ veterans focuses on urban populations, it is important to examine patient and provider perspectives to better understand what is common and different about the experiences and needs of rural and urban dwellers (15). In the current study, we define urban and rural based on where the veterans who were served by the facilities lived (e.g., urban-dwelling vs. rural-dwelling).

To increase health equity, the VHA has taken several steps to improve healthcare for LGBTQ+ veterans. In 2012, VHA created a national LGBTQ+ Health Program and currently has two specific directives for LGBTQ+ affirming care: VHA Directive 1341 (Providing Health Care for Transgender and Intersex Veterans) and VHA Directive 1340 (Provision of Health Care for Veterans Who Identify as Lesbian, Gay, Bisexual, or Queer). LGBTQ+ affirming care is healthcare that is inclusive and understanding of the unique healthcare needs of LGBTQ+ people. These directives provide policy and guidance on how to provide LGBTQ+ affirming care and treat all LGBTQ+ veterans with dignity and respect. For example, the directives offer information on how to use a veteran’s appropriate name and pronouns and refer to care and the frequency in which sexual health assessments should be conducted. These policies also prohibit harmful sexual orientation and gender identity and expression change efforts or so called “conversion” therapy practices. The delivery of LGBTQ+ affirming care benefits LGBTQ+ veterans by reducing barriers to access and utilization in VHA, increasing comfort among medical providers, and encouraging patients to disclose their health issues, thus leading to improved healthcare and mental and physical health (24, 25).

Although these policies are in place, there is limited data on their implementation in VHA healthcare practice from the perspectives of providers and veterans. The goal of this project was to (1) document provider understanding of and LGBTQ+ veterans experience of LGBTQ+ affirming care in VA, (2) identify barriers and facilitators associated with provider delivery and LGBTQ+ veteran experience of LGBTQ+ affirming care, and (3) assess variations in provider understanding and LGBTQ+ veteran experience of LGBTQ+ affirming care in rural versus urban settings. This manuscript reports findings from qualitative interviews with providers and LGBTQ+ veterans, analyzing their perceptions of barriers and facilitators to accessing, delivering, and receiving LGBTQ+ affirming care in two VHA medical centers (VAMCs) and one VHA community-based outpatient center (CBOC) in the southern United States.

## 2 Materials and methods

One-time qualitative interviews were conducted with VHA providers and LGBTQ+ veterans. Constructs from the Consolidated Framework for Implementation Research (CFIR), adapted to include three health equity domains (i.e., culturally relevant factors, clinical encounter, and societal context), informed data collection and analysis (26–28). Incorporating the health equity domains into CFIR allowed for the specific measurement of health inequities for LGBTQ+ veterans and were used design interview questions and guide analysis. Provider interviews focused on their awareness, understanding, and experience of providing LGBTQ+ affirming care. LGBTQ+ veteran interviews focused on their experiences receiving LGBTQ+ affirming care at VHA. Interview data were analyzed using template analysis. All research activities were approved by the Central Arkansas Veterans Healthcare System Institutional Review Board and conducted following the Declaration of Helsinki as revised in 2013.

### 2.1 Setting

This study was conducted within the VHA. Potential participants were identified at three VHA facilities: one urban medical center, one rural medical center, and one rural CBOC. The urban medical center was in southeast Louisiana and the rural medical center and CBOC were both located in Arkansas. In VHA, medical centers offer a wide array of inpatient and outpatient services, including surgery, critical care, mental health services, radiology, and physical therapy. The CBOCs typically only offer limited outpatient services (e.g., primary care, outpatient mental health) in locations that are geographically distinct from the medical center. Each CBOC has a “parent” medical center and most medical centers have multiple CBOCs.

### 2.2 Framework

CFIR is an implementation determinants framework, meaning it was designed to identify factors believed or empirically shown to influence implementation (28). Five major domains comprise CFIR, including intervention characteristics, outer setting, inner setting, characteristics of the individuals involved in implementation, and implementation (28). There are several constructs within each overarching domain. For the current study, we examined the outer setting, inner setting, and individual-level characteristics. Specifically related to the outer setting, we examined patient needs and resources and external policies and incentives. Related to the inner setting, we examined culture, implementation climate, learning climate, available resources, and access to knowledge and information. Finally, regarding the characteristics of the individual, we examined knowledge and beliefs about the intervention (i.e., LGBTQ+ affirming care as indicated by two VHA policies), individual stage of change, and other personal attributes (e.g., tolerance of ambiguity, intellectual ability, values).

Per recommendations by Woodward and colleagues, we enhanced the CFIR domains with the three health equity domains identified in the Health Equity Implementation Framework (27). These include culturally relevant factors, clinical encounter, and societal context. Culturally relevant factors of recipients include characteristics of people affected by the implementation effort (e.g., socioeconomic status, race and ethnicity, language, health beliefs, or trust in providers). The clinical encounter refers to the transaction between patients and providers during healthcare appointments. Providers’ and patients’ verbal and nonverbal behaviors shape the clinical encounter. The interactions during this encounter influence decisions about diagnosis and treatment and how care is delivered. Finally, the societal context includes economics, physical structures, and sociopolitical forces (e.g., structural discrimination from institutions, state level laws, political beliefs).

### 2.3 Participants and procedure

Providers were recruited through snowball sampling across services (e.g., primary care, mental health, audiology, infectious disease). We began by asking the LGBTQ+ veteran care coordinator for the names of providers who worked with LGBTQ+ patients and who they believed would be willing to speak with us about their experiences providing LGBTQ+ affirming care at each of our identified sites. We then asked this initial set of providers to suggest their peers with variability in awareness, understanding, and use of LGBTQ+ affirming care (“Would you be willing to provide some names of providers who we could talk to? Can you think of any providers who may approach LGBTQ+ affirming care differently than you do?”). We then contacted potential participants based on the recommendations we received. Providers were VHA employees completing the qualitative interviews during their work duty time, so they could not be compensated for their participation.

Inclusion criteria for providers included (1) being employed as a VHA provider and (2) treating LGBTQ+ veterans. Potential participants were scheduled and screened for eligibility before completing the informed consent process. After completing this process, providers were asked what service they worked in and their discipline. Qualitative interviews were conducted virtually with the option of visual or audio conferencing and were 30 min long. Providers were interviewed between July and December 2021.

LGBTQ+ veterans receiving VHA care were identified through a one-time chart review of veterans previously enrolled in LGBTQ+ specific programming (e.g., support groups) or with a diagnosis often associated with LGBTQ+ identities (e.g., gender dysphoria, high-risk homosexual behavior). At the time of the study, VHA did not have available fields for self-reported sexual orientation and gender identity in the electronic health record. Therefore, these diagnoses served as a proxy measure of LGBTQ+ identity. Of note, patient-reported sexual orientation and gender identity are the gold standards for identifying LGBTQ+ people (4). If participants agreed to participate, they were asked to self-identify their sexual orientation and gender identity at the time of the interview. The identified veterans were recruited via opt out letters. These letters were modified from opt out letters that were used to contact Veterans with recent suicidal ideation and attempts (29, 30). Additionally, we asked participants about the acceptability of these letters, and they found them to be acceptable. Table 1 lists ICD-9 and ICD-10 codes used to send opt out letters to veterans. The letters stated that if they did not call to opt out in 2 weeks, they would receive a phone call from researchers asking them to participate in the study. LGBTQ+ veterans received $50 for participating in the study.

**Table 1:** ICD-9 and ICD-10 codes used to potentially identify LGBTQ+ veterans.

ICD-9 Codes		ICD-10 Codes	
302.85	Gender identity disorder in adolescents or adults	F64.0	Transsexualism
302.6	Gender identity disorder not otherwise specified	F64.1	Dual role transvestism
302.5	Transsexualism	F64.2	Gender identity disorder of childhood
302.3	Transvestic fetishism	F64.8	Other gender identity disorders
302.0	Ego-dystonic homosexuality	F64.9	Gender identity disorder, unspecified
302.52	Trans-sexualism with homosexual history	F65.1	Fetishistic transvestism
		Z87.890	History of sex reassignment surgery
		Z72.52	High risk homosexual behavior
		Z72.53	High risk bisexual behavior

It is important to note that as proxy measures, the ICD 9 and ICD 10 codes, are outdated and include harmful language. Gender identity disorder and transsexualism are no longer appropriate terms to use when describing people who are transgender and gender diverse (31). Gender identity disorder suggests the experience of being transgender is a disorder and transsexualism is an older term derived from obsolete medical beliefs that conflate gender identity and sexual orientation (7). The use of transvestism stigmatizes people who do not conform to gender stereotypes or within the gender binary (7). Finally, the term homosexual is considered an antiquated clinical term derived from medical gatekeeping and a misunderstanding of the LGBTQ+ community (31). Although we used these codes initially to identify veterans to recruit by mailing opt out letters, we did ask them if they self-identified as part of the LGBTQ+ community and to list their identities.

Inclusion criteria for LGBTQ+ veterans included (1) self-identifying as part of the LGBTQ+ community and (2) taking part in any VHA healthcare visit in the last 3 months. During the phone call to screen and recruit veterans, interested candidates were instructed to answer “yes” or “no” to the following questions (1) Do you identify as LGBTQ+? and (2) Have you had a VHA healthcare visit in the last 3 months? Eligibility required an answer of “yes” to both questions. Candidates were either scheduled to complete the 30–60 min phone interview or offered the opportunity to complete it at that time. At the start of the interview, Veterans were then asked, “How would you describe your gender identity?” and “How would you describe your sexual orientation?” to document their self-described sexual orientation and gender identity at the time of the interview. LGBTQ+ veteran interviews were conducted between October and December 2021.

### 2.4 Qualitative interviews

The CFIR-informed interview questions provided by framework developers for public use were used for semi-structured interview guide development. Questions were tailored to be specific to LGBTQ+ affirming care by the investigators and informed through consultation with other investigators who have conducted LGBTQ+ research with providers in civilian samples (22). See Table 2 for examples of interview questions.

**Table 2:** Example interview guide questions by participant group and CFIR domain.

Participant group	Domain	Subdomain	Example question
Provider	Outer setting	Patient needs & resources	How familiar do you think other providers at your VA are with LGBTQ+ veteran issues?
			What evidence do you see of LGBTQ+ affirming care at your facility?
			Have you heard any stories about the experiences of LGBTQ+ veteran patients at your VA?
		External policies & incentives	What kind of guidance is available to you regarding providing LGBTQ+-affirming care?
LGBTQ+ veteran	Outer setting	Patient needs & resources	Do you see any evidence that employees at your VA are LGBTQ+ affirming?
			*Probe (if needed):* What do you see? What do you wish you saw?
			What has your experience as an LGBTQ+ veteran been like receiving care at this VA?
			What have you heard from other LGBTQ+ veterans regarding the care they receive at your VA?
	Inner setting	Culture	How would you describe the openness and acceptance of the provider you saw during your last visit towards LGBTQ+ veterans? *Follow up:* The openness and acceptance of providers in general at your VA?
			Can you think about anything about your VA that makes it easier or harder to receive care as someone who is [Veteran’s self-described sexual orientation and/or gender identity]? (urban vs. rural, provider factors, staff factors?)

### 2.5 Data analysis

All interviews were recorded, transcribed verbatim, and rapidly analyzed using template analysis (32). The version of the approach used in this study is a data reduction technique developed by health services researchers (32). Template analysis is useful when there is a relatively short turnaround for information to inform implementation in health services settings. Template analysis involves developing a template aligned with the interview guide and includes potential domains, subdomains, and categories. The lead analyst (RSS) reviewed interviews and developed domains, subdomains, and categories based on interview content. Domains and subdomains were based on the overall CFIR domains (e.g., the domain of characteristics of the individual and the subdomain of knowledge and behaviors). Categories were developed based on participant responses (e.g., uncertainty in delivering LGBTQ+ affirming care). Although domains, subdomains, and categories were developed *a priori* based on the interview guides, they were modified as the analysis was conducted.

The lead analyst then reviewed interview transcripts and summarized the content gathered from the interviews in a template. A senior analyst and expert in template analysis (THA) consulted on this project. The senior analyst synthesized content from individual templates to achieve consensus, provided feedback during template development, piloting, and analysis, and audited every fourth template. Upon completion of templating all interviews, the lead analyst created a template matrix that included domains, subdomains, and categories which could be viewed by site and by participant to examine differences across sites and individuals.

## 3 Results

Of the 32 providers identified, 11 participated in the study. Twenty-one providers across all three sites either did not respond or declined. Reasons for provider declination included (1) reporting that they did not see LGBTQ+ veterans, (2) time constraints, and (3) affirmation that they already provided LGBTQ+ affirming care and did not think they needed to participate. Of the 73 veterans identified, 12 participated in the study. Two veterans called to opt out of participation. One noted they were not out publicly yet and did not wish to participate. Another left a message stating they were not interested. Four opt out letters were returned as undeliverable. Table 3 shows demographic data for VHA provider participants and LGBTQ+ veteran participants, respectively.

**Table 3:** LGBTQ+ veteran and provider participant demographics.

LGBTQ+ veterans		*n*
Age	Median Age: 48 (Range 27–74)	
Race	White	8
	Black/African American	3
	American Indian	1
Gender	Transgender women	6
	Transgender men	3
	Cisgender men	3
Sexual orientation	Gay	1
	Lesbian	1
	Bisexual	1
	Pansexual	1
	Unsure	1
	Straight	5
	Attracted to emotional intelligence	1
	No response	1
Facility	Urban VAMC	5
	Rural VAMC	5
	Rural CBOC	2
Providers		
Role	Physician	6
	Psychologist	1
	Speech pathologist	1
	Social worker	3
Service	Audiology	1
	Mental health	2
	primary care	4
	Primary care mental health	1
	Women’s health	3
Facility	Urban VAMC	5
	Rural VAMC	5
	Rural CBOC	1

Results are grouped into barriers and facilitators to LGBTQ+ affirming care. We present a full overview of barriers and facilitators in Supplementary Tables S1, S2, respectively. Barriers that converged between providers and veterans included (1) providers lack experience in delivering LGBTQ+ affirming care, (2) non-affirming institutional structures within VHA, and (3) societal discrimination impacts what LGBTQ+ veterans expect at VHA.

### 3.1 Barriers to LGBTQ+ affirming care

Provider and veteran responses were categorized into barriers to delivering and receiving LGBTQ+ affirming care. We elaborate on categories where the reports of providers and veterans converged, including providers’ lack of experience in delivering LGBTQ+ affirming care, non-affirming institutional structures within VHA, and concerns about societal discrimination.

#### 3.1.1 Providers lack experience in delivering LGBTQ+ affirming care

Approximately half of the providers admitted they felt unprepared to deliver LGBTQ+ affirming care, as the topic was not integrated into their previous medical education. The providers who reported feeling unprepared were based in primary care and one in women’s health. When asked about this topic, one provider readily disclosed, “I was immediately thinking my blood pressure just went up about twenty points because honestly, I feel extremely unprepared.”

Similarly, all the LGBTQ+ veterans reported a wide variability in their healthcare experience; some reported no issues and high satisfaction with their care, and others reported experiencing discrimination or a general lack of knowledge by their VHA providers. One veteran explained,

“I have to remind my [Department of Veterans Affairs] (VA) doctor that I need to have blood work done or get my results from my endocrinologist. I have to remind them that I need to have my labs drawn …. then it’s like an afterthought of, ‘Oh, yes, I guess you do need this refill.’ Or ‘Oh, yeah, we better request those records.’”

Several transgender veterans wished their providers in VHA healthcare settings were better versed in and able to share accurate information about the physical and psychological aspects of medical transition.

#### 3.1.2 Non-affirming institutional structures within VHA

A majority of providers attributed other barriers to delivering LGBTQ+ affirming care to difficulties related to VHA as an institution. Broader categories pertaining to the institution of VHA include “VA culture” and deficiencies associated with the electronic health record, which at the time of data collection did not have sexual orientation and gender identity data available to providers. Without easy access to this information in the electronic health record, providers could not rely on conducting chart reviews to adequately identify and address the needs of LGBTQ+ veterans.

A few providers also discussed the formal culture of the VA. For example, there is an expectation that one should be referred to by honorifics often associated with perceived gender (e.g., Ms. and Mr.). One provider in Women’s Health described coaching another provider caring for a transgender man. The participant said the other provider habitually used “Ms.” as a women’s health provider and continued accidentally misgendering the patient.

“I had one issue with one provider [misgendering a transgender man] but she knew when she was doing it, and the more she tried to correct herself with the pronoun, the worse it got. She was so upset with it that she picked up the phone afterwards and called me to talk to me about it. And we continued to work with her on that because it’s a habit.”

A majority of providers discussed concerns about LGBTQ+ veterans not feeling welcome and affirmed at VA. One provider described a patient who did not disclose his sexual orientation and HIV status out of worry of negative stereotypes.

“I had a patient who passed away recently. He’d been my patient forever, and he had HIV and for years did not tell me. He got his treatment outside the VA. He used to hide it because he worked at the VA years ago. He was worried that if that ever got into his chart, people would associate it with negative stereotypes about his sexual preferences.”

Providers described structures within VHA that were not designed for LGBTQ+ veterans. For example, if a transgender man with anatomy commonly ascribed as female was seeking care through the women’s clinic, this man automatically “outed” himself when sitting in a waiting room designed specifically for women. Providers also voiced concerns that LGBTQ+ veterans would not be comfortable being open and honest with their providers due to anticipated and actual discrimination within the VHA and society in general (as discussed below). In this vein, a veteran raised concerns about presenting at the VHA and the potentially detrimental consequences of entering the VHA or military-related functions as a transgender veteran.

“If a person walks into the VA and they do not pass … that makes the facility dangerous. It could put the veteran at risk from other veterans, and I say that because many people within the veteran community are very vocal about trans people even serving in the military. It’s like we do not exist…. If you flub up, like your presentation is not as good, or your voice is not as clear, or maybe something happens, that can put you in danger. And if you are in the wrong environment with the wrong people, especially with males to females, that’s how trans people get beat to death, beaten in a parking lot, and it happens. That happens every day. I’ve had negative experiences at military veteran-related functions.”

The veteran’s experience highlights how what happens outside of VHA can directly translate to concerns about what to expect at VHA. If an LGBTQ+ veteran encounters an unsafe environment in an event or area where other veterans are present, the worry about the VHA being similar is present.

Veterans struggled with institutional policies or infrastructure that reinforced discrimination against LGBTQ+ veterans. For example, one veteran discussed difficulty finding a gender-neutral bathroom when visiting the VHA. Another veteran described how they had been denied care at the VHA in their home state and forced to drive hours to receive hormone therapy due to a lack of providers willing to deliver it.

“They were forcing me to go to [another city in another state]. They said there was no other trans hormone doctor in Louisiana, but I knew there was. They were making me go to [city] and when I went there, it wasn’t even a good experience. The doctor came in and said, ‘You want to be a man. Here, take these to be a man.’ That was the full interaction.”

A majority of the transgender veterans who were interviewed stated institutional barriers made it difficult for them to access care or want to access care at VHA. Another discussed the bureaucratic burdens of getting his name changed on all his VHA records and forms. This veteran described the additional frustration of knowing he was being turned away by clerical staff because he was transgender. He described being told if he were changing his name due to marriage, they could assist him. However, he stated that because he was changing his name on court documents to reflect his current gender identity, he was turned away despite being against VA policy. The veteran wanted someone who could assist him in overcoming the administrative hassles or explain all the pieces of paperwork necessary to update his name in the VHA system.

#### 3.1.3 Societal discrimination impacts what LGBTQ+ veterans expect at VHA

Veterans and providers discussed how discrimination outside of VHA can impact what LGBTQ+ veterans expect to experience inside VHA. Further, providers and LGBTQ+ veterans discussed concerns about structural discrimination through anti-LGBTQ+ laws and policies. Although these policies would not apply to VHA, they reported concerns about how these laws and policies influence access to care. One provider discussed that even when providers were affirming, it would be reasonable that LGBTQ+ veterans may be hesitant to disclose information about their sexual orientation or gender identity due to societal discrimination in general.

“Based on the remarks and judgment and harassment they feel in general in the community may make them less comfortable or less willing to disclose things about their identity to their provider or even come here to seek care. Harassment can happen on their bus trip here or outside [of the VA].”

Similarly, one veteran discussed the detrimental effects of societal discrimination on transgender veterans. She noted the concerns transgender veterans may have due to a history of real violence towards themselves or towards transgender communities in general.

“[Transgender veterans] feel isolated, and some of them live in fear of being hurt. At one time or another they were mistreated, but I think if somebody does not like them, they’ll wait for them to corner them if you know what I mean.… If you look at it in society there’s been a lot of violence toward LGBT [people].”

Although the experiences described were not directly at VHA, both providers and veterans highlighted how they can impact the willingness to seek VHA care. If a veteran encounters discrimination in society in general, they are likely going to perceive that discrimination will also occur within VHA.

### 3.2 Facilitators of LGBTQ+ affirming care

We elaborate on categories in which provider and veteran reports converged, including provider interest’s impact in delivering LGBTQ+ affirming care and acceptance towards LGBTQ+ veterans. Some key differences by group noted by several participants include consultation with trusted peers (providers) and advocating for and awareness of one’s own healthcare needs (veterans).

#### 3.2.1 Positive impact of providers invested in delivering LGBTQ+ affirming care

A majority of the providers interviewed expressed a desire to build their skills to delivering LGBTQ+ affirming care. One provider explained, “Even before we talked today, [I] have had a strong interest in more educational development about this community and even maybe some interest in doing more with the hormone therapy.” A second provider elaborated, “Anything I can do to help out with this particular population has personal meaning for me, but also just in general, it’s a good thing to provide care to people who for a long time have not gotten the care that they need.” Even with personal reasons and trusted peers as resources, providers still reported aspirations to increase their education in LGBTQ+ affirming care. Almost all providers wanted more education and training to enhance care for LGBTQ+ veterans. However, they also described time constraints as preventing them from participating in training and, if they were able to attend such training, to then become practiced at applying the new knowledge and skills they learned.

Relatedly, veterans clarified the impact of knowing that providers care and were working towards providing LGBTQ+ affirming care. One veteran appreciated the proactiveness of the care team coordinating her hormone therapy, “They’re not just [saying] come see us when you need care. They make sure you are okay, and they do the right things…. My last visit was with [hormone therapy doctor]. Her and her nurses are fabulous.” Another veteran reported generally being treated well by his providers and not experiencing heterosexism. “Because I’m openly gay and any healthcare provider I’m dealing with where it might be a relative issue, I am very forthright about it and I’ve never seen any kind of negative reaction at all.” Nevertheless, several LGBTQ+ veterans suggested recommendations for VHA to improve LGBTQ+ affirming care, such as organizing support and educational groups. Veterans also requested a desire for more knowledgeable providers.

Veteran participants clarified that the range of medical services they could obtain from the VHA were limited, observing that VHA does not provide gender-affirming surgeries at this time. One veteran hoped this would change soon and that if other federally-funded healthcare options (e.g., Medicaid) allow for gender-affirming surgeries, the VHA should also.

#### 3.2.2 Openness and acceptance towards LGBTQ+ veterans

In addition to delivering and receiving specific types of care, providers and veterans emphasized the importance of openness and acceptance as part of high-quality LGBTQ+ affirming care. Some providers described their own experiences as belonging to the LGBTQ+ community or valuing social justice during the interviews. One provider described how the facility responded to LGBTQ+ veterans,

“I think that our facility is very open and accepting. I think that the providers do not pass any kind of predetermined judgment…. I think that as a group we are all very open and we have many staff, many residents, many employees that belong to that community as well. So, I think as a group in general we are very accepting.”

Similarly, veterans discussed the importance of acceptance received from providers and staff. One veteran stated, “They are open, accepting, and understanding… for the most part, I have not had any issues gender-wise. I have had the utmost respect.” A veteran who was a cisgender, pansexual man stated, “I feel they are open and accepting. I feel like they do a good job.” Veterans emphasized the importance of being affirmed and seen as they are in their healthcare environment. These experiences of being accepted and affirmed reportedly increased the likelihood that these veterans would continue to access VHA care. In interviews, veterans highlighted that providers who truly understood and provided LGBTQ+ affirming care enhanced their trust and engagement in healthcare at VHA.

#### 3.2.3 Providers are comfortable consulting with trusted peers

Providers discussed the power of personal connection and resources. For example, several providers referred to a previous clinic director who identified as a transgender woman. Although this director no longer worked in this clinic, the providers reported feeling comfortable contacting her for support. Providers also mentioned key contacts within their own VHA system, including the LGBTQ+ veteran care coordinator, transgender care coordinator, and women’s health service leads to name a few. Providers expressed comfort reaching out to colleagues they knew possessed subject-matter expertise in LGBTQ+ affirming care. This willingness to seek support from trusted colleagues underscores the importance of professional resources and consultation within the VHA system.

#### 3.2.4 Veterans as knowledgeable about and able to advocate for their healthcare needs

Although veterans expressed frustration about knowing more than their doctors, they also discussed how it is helpful for patients to understand their own healthcare needs. “If I could give a veteran who was coming out as transgender advice, I would tell them to do the research on everything: blood levels, the effects of hormones, how to mentally transition first before your body, specifically that mental transition first then seek medication. But to learn everything first because more than likely they need to know everything. They need to know more than the average doctor would know.”

Similar to knowing their own healthcare needs, veterans emphasized the importance of advocating for their own needs and responsiveness from providers. One veteran stated, “I knew there was a trans doctor at the VA, but they would not let me go to [closest VA]. So, I ended up looking online and finding her. I requested to meet with her.… She actually called me at six o’clock in the morning and said, ‘I’ll be your doctor.’ So, I did not have to go to [another city in another state] anymore.”

The three veterans who were cisgender men with sexually minoritized identities all received pre-exposure prophylaxis (PrEP) at VHA. They were all able to speak to their needs of accessing PrEP to reduce the risk of getting HIV. One veteran stated, “I feel like anyone who has sexual relations with anybody should have HIV checks more regularly. I try to get one at least every 2 to 3 months. If I do not personally go on my own, [the HIV test] gets done when I get my PrEP with my blood drawn. They also check for [sexually transmitted diseases]. If most people know [their sexual health] it protects others.”

### 3.3 Recognizing intersecting identities

In addition to examining overall barriers and facilitators, we were interested in differences based on living in an urban vs. rural setting. Participants discussed differences based on the locations in which they received healthcare. For example, one cisgender, gay male participant who accessed care at the urban VAMC stated, “I think a factor for the providers and staff is that New Orleans has historically been very accepting situation for that community. [LGBTQ+ issues are] something that if you live in that region, you have to deal with it. It’s not a closeted community.” In contrast, a transgender woman accessing care at a rural CBOC stated,

“I happen to be living in Arkansas, which is bible belt territory and if I’m more openly presenting – Say I dress very feminine and have facial hair because ladies can have a beard too – if I’m running around all femmed up with a beard down to my Adam’s apple, I’m probably going to get my ass beaten simply for being in the wrong place because it’s 50 years behind here.”

Providers also spoke about locational differences. For example, one provider at the rural VAMC commented, “I think in states that are less accepting, like Arkansas, there’s the danger of knowing what is an affirming space […] I think for someone who is trans and without very clear spaces, it can be scary, and people can feel hypervigilant to danger if they aren’t sure who is going to say or do something.” Another provider discussed the difference between the urban VAMC and living in the urban city: “I would imagine in my mind that people coming to the VA would probably feel more comfortable in New Orleans just because I feel like people can be a little more free to be themselves [here].”

In addition to rurality, considerations about the intersection between LGBTQ+ identity and race were discussed by veterans as well. For example, one veteran noted attending LGBTQ+ support groups but realized he was the only Black person who attended. He stated he asked another Black LGBTQ+ veteran why they did not attend, and the response was, “It’s because they did not really see people like themselves really doing it. The providers were all white people.” Veterans reported a desire for community, with several noting that LGBTQ+ support groups were limited due to the COVID-19 pandemic or there was limited representation in the LGBTQ+ support groups.

Related to race, veterans called attention to how they may experience anti-LGBTQ+ discrimination in diverse racial and ethnic communities, including communities to which they may or may not belong. The experience of anti-LGBTQ+ discrimination from different marginalized groups (e.g., rural-dwelling people, racial and ethnic minoritized people) highlights how although people may share some aspect of similarity in identity or even both belong to marginalized groups, they can experience discrimination of their additional marginalized identities. Societal discrimination can be pervasive through all people resulting in further discriminating and harm.

## 4 Discussion

LGBTQ+ affirming care is crucial for LGBTQ+ veterans to comfortably access and remain engaged in VHA care. The main goal of this paper was to understand the implementation determinants of LGBTQ+ affirming care at two VAMCs and one CBOC in the southern United States. Applying an enhanced CFIR to organize and interpret our findings, we identified barriers and facilitators within the outer setting, inner setting (VHA), individual characteristics, and health equity domains (i.e., culturally relevant factors, clinical encounter, societal context).

Although VHA’s LGBTQ+ affirming care policies are in place, VHA providers and LGBTQ+ veterans described a lack of overall awareness regarding the policies and how to provide or access healthcare optimally aligned with the policies. However, providers reported a strong desire to learn more and enhance care for LGBTQ+ veterans, while LGBTQ+ veterans wanted providers to be more aware of their unique needs and experiences. Of note, few providers referenced the numerous VA online training materials in LGBTQ+ health or the national provider-to-provider transgender e-consult as tools that they used for education and training, suggesting that more widespread dissemination is needed. VHA’s LGBTQ+ affirming care policies state that care for LGBTQ+ veterans should be offered in VHA facilities. Although care may be offered, both veterans and providers discussed gaps in providers’ knowledge and ability to deliver high-quality care for LGBTQ+ patients. For example, being turned away for care that was not available (i.e., hormone therapy). Notably, veterans were satisfied with care when their providers were open and affirming. This finding dovetails with previous research indicating that LGBTQ+ people may be satisfied with care if providers make some effort to affirm their identity (even if it is not perfect or care is suboptimal) (33, 34).

Results are similar to evaluations of LGBTQ+ affirming care implementation outside of VA. For example, providers are interested in more learning and training and find it difficult to find the time to attend training (20). Civilian LGBTQ+ affirming care implementation studies found concerns over the messages the system sends and how these may impede LGBTQ+ affirming care (e.g., unenforced policies, physical structures not designed for LGBTQ+ patients) (20, 35). Our results support VHA and civilian concerns from providers about comfort, how to provide LGBTQ+ affirming care, and desires for more training. LGBTQ+ veteran concerns about VHA being a welcoming environment were also echoed in the current study.

The results align with previous studies examining VHA providers’ experiences of working with LGBTQ+ veterans and LGBTQ+ veterans’ perceptions of VHA care (36, 37). The current study’s findings support literature suggesting LGBTQ+ veterans experience ambivalence and reluctance to seek treatment at VHA due to experiences with other veterans and the military (11). Veterans discussed this related to discrimination within VHA and society in general.

Veteran and provider reported experiences of societal discrimination and discrimination within VHA, as well as the interaction between the two, speak to how LGBTQ+ veterans may experience minority stress and how their environment can exacerbate it. For example, if an individual encounters discrimination in the community while walking to VHA or using public transportation, it likely will make them even more on guard when they enter the facility. They may begin to anticipate discrimination from other veterans, staff, or providers as they enter their healthcare environment. This compounding effect of minority stress can negatively impact their physical and mental health. It may be helpful to improve LGBTQ+ affirming care throughout the entire VHA system to reduce minority stress. LGBTQ+ affirming care includes affirming actions of providers and staff (e.g., using correct name and pronouns, asking about partners in a gender-neutral manner) as well as the environment (e.g., safety signals, materials with same-gender couples).

Results of the current study contribute additional information to the literature on provider- and patient-level barriers to LGBTQ+ affirming care. Further, these results add helpful information to consider intersecting identities. For example, veterans and providers noted the difficulties of living as an LGBTQ+ veteran in rural settings. Veterans who were transgender and lived in rural areas reported differences from those who were cisgender and lived in urban areas. Given that much of the research on LGBTQ+ affirming care happens in urban environments, the current study provides some unique considerations to the situation of rurality and living as an LGBTQ+ veteran. For instance, providers and clinics in rural settings may need to pay even more attention to LGBTQ+ affirming care. This could include ensuring safety signals are in major entryways and patient exam rooms so that LGBTQ+ veterans know the location is safe and affirming. Making healthcare settings more inclusive could also include providing more training to providers in rural settings to ensure they can provide LGBTQ+ affirming care as well as be thoughtful to the environment LGBTQ+ veterans may be experiencing.

In addition to rurality and gender, we observed difficulties for participants who identified as transgender and part of an ethnic and racial minoritized group. It may be important to consider the needs for veterans and their many identities in designing services that are made for or designed by people with multiple shared identities (e.g., peer support groups, providers who themselves who share similar characteristics to veterans). If people cannot create or offer services specifically based on identity considerations, then providers should consider their own positionality and ways to make veterans feel welcome and included. Additionally, the LGBTQ+ community is not a monolith and includes multiple genders and sexual orientations (e.g., non-binary people, bisexual people). Therefore, providers and staff should consider patients’ specific identities versus generalizing information about LGBTQ+ people overall.

In this study, several barriers to delivering LGBTQ+ affirming care (providers) and receiving LGBTQ+ affirming care (veterans) were identified. VHA currently has two specific policies focused on providing LGBTQ+ affirming care – one for transgender and gender-diverse veterans and one for LGBQ+ veterans. Policies are an effective initial step to creating an inclusive and affirming environment for LGBTQ+ Veterans. However, policies alone do not create change. Mechanisms need to be in place to facilitate dissemination, awareness, and enforcement. Without specific mechanisms for implementation and accountability to these policies, change will not occur. There are certain mechanisms already in place at the national level (e.g., the LGBTQ+ veteran care coordinator program (38), national availability of trainings), and more mechanisms are needed.

Results from this paper can support additional work to improve the implementation of these policies. The next steps after identifying barriers would be to purposefully identify implementation strategies (i.e., activities within a setting to address barriers and ideally assist with effective implementation) to improve the implementation of the LGBTQ+ affirming care health policies. These strategies may target the healthcare system, providers and staff, and LGBTQ+ veterans. Regarding strategies that face the healthcare system, developing relationships between several invested key personnel (e.g., network directors, facility directors, LGBTQ+ veteran care coordinators) may be a successful strategy to address structural and systems-level barriers to improve LGBTQ+ affirming care. Examining and evaluating LGBTQ+ affirming care at the systems level may also be beneficial to identify how many LGBTQ+ veterans are accessing VHA care and whether their needs are being met.

Participants clarified the need for more education and training for providers and staff. Some providers noted this during their interviews and included providers’ willingness to learn (e.g., the provider in Women’s Health who recognized she was misgendering someone and reached out for support). The potential need for more education and training was also gathered from provider declination to participate. For example, one provider declined to participate because they said they do not treat LGBTQ+ veterans and another declined because they felt they already provide LGBTQ+ affirming care. These suggest maybe a limited awareness of LGBTQ+ veterans and the changing and evolving nature of their needs. Educational meetings to provide training and resources would be beneficial, including protected time for participants to attend and practice what they have learned (20). Despite the availability of two dozen trainings in the VA online education portal, this resource was only mentioned by a few providers. It may also be possible to change the infrastructure so that providers and staff are required, at minimum, to learn the basics of LGBTQ+ affirming care, including inclusive language practices to prevent misgendering. For example, certain VAMCs mandate that all providers and staff complete basic online training in LGBTQ+ affirming care. Such a mandate could be broadened to other VHA facilities. It may be helpful to design specific tools (e.g., pocket cards) and focused education (e.g., fast facts during team huddles) that can be quickly and efficiently delivered to overcome time constraints shaping providers’ workdays.

Providers also brought up consulting trusted peers. Although this is a facilitator, it may place undue burden on a handful of people to be the only local LGBTQ+ affirming providers. Meanwhile use of nationwide provider-to-provider transgender e-consultation was not mentioned. Recognizing the trusted and expert peers may be a helpful building block to leverage for further education and create greater capacity within healthcare systems.

Finally, LGBTQ+ veterans in this study provided several recommendations related to how they would like to receive care. All the veterans reported knowledge of their own healthcare needs as LGBTQ+ veterans. Typically, policies are not implemented or considered with those most affected by them in mind (39). It is possible that substantively including LGBTQ+ veterans in policy implementation may increase the likelihood that policies are implemented with their needs in mind and reach those most in need of the policies (39). Practically, this could include allowing veterans to provide input on policy implementation strategies, materials, and resources. Co-designing the policy implementation resources or creating resources with LGBTQ+ veterans may allow more collaborative power sharing with LGBTQ+ veterans as well as integrate their lived experience into implementation. This would allow for veterans’ points of view and needs to be centered in the work of policy implementation.

### 4.1 Limitations

We attempted to recruit providers with diverse viewpoints, but the majority recruited for this study were highly invested in LGBTQ+ affirming care. Although we recruited a couple of providers with limited LGBTQ+ affirming care knowledge, it would be helpful to recruit providers who were opposed to or had extremely limited knowledge related to LGBTQ+ affirming care. Further, we did not collect demographic information from providers beyond their work setting and role. Another limitation is using diagnoses as a proxy measure to identify potential participants because these diagnoses can be stigmatizing as well as inaccurate in identifying LGBTQ+ people, as described above. It is best practice to use self-identified gender identity and sexual orientation to identify LGBTQ+ people (4). This was not possible at the time of this study as the gender identity and sexual orientation data fields only recently became available in VHA. It is likely that accurate identification of LGBTQ+ veterans in the electronic health record (instead of using proxy measures of ICD codes) will allow for more effective recruitment of these veterans for research in the future. Additionally, we were not able to recruit any people with genders outside of the binary and were unable to recruit any cisgender women; therefore, various gender experiences as well as sexual minoritized people are underrepresented in the sample.

### 4.2 Conclusion

Both LGBTQ+ veterans and VHA providers reported a desire for more LGBTQ+ affirming care in VHA. Veterans also felt affirmed and welcomed when they believed they received LGBTQ+ affirming care. Veterans provided several recommendations for what they would like to see within VHA. Engaging LGBTQ+ veterans in determining how to improve their healthcare may be a promising path forward to increase understanding to LGBTQ+ affirming care policies as well as ultimately improve access to and reduce inequitable healthcare for LGBTQ+ veterans. Creating collaborative structures can enable LGBTQ+ veterans and VHA personnel to work together to implement these healthcare policies. Using the information in this study may be one solution that increases the effective implementation of LGBTQ+ affirming care in VHA.

## Data availability statement

The datasets presented in this article are not readily available because they are qualitative data specific to a marginalized population. Requests to access the datasets should be directed to the corresponding author.

## Ethics statement

The studies involving humans were approved by Central Arkansas Veterans Healthcare System. The studies were conducted in accordance with the local legislation and institutional requirements. The ethics committee/institutional review board waived the requirement of written informed consent for participation from the participants or the participants’ legal guardians/next of kin because This study required interviews conducted via telephone.

## Author contributions

RS, SL, JK, TA, MK, and JS assisted with conception and design of the project. PM provided feedback on the project, assisted with site identification, and recruitment of provider participants. CW provided feedback on measurement tools. RS and TA performed the qualitative analyses. RS wrote the first draft of the manuscript. SL and CW wrote sections of the manuscript and heavily revised initial drafts. All authors contributed to manuscript revision, read, and approved the submitted version.
