# Supplementary material for: Implementation of LGBTQ+ affirming care policies in the Veterans Health Administration: preliminary findings on barriers and facilitators in the southern United States
Source: Front Public Health. 2024 Jan 30;11:1251565. doi: 10.3389/fpubh.2023.1251565 (PMC10861648; doi:10.3389/fpubh.2023.1251565)
Supplement: Supplementary file 1 [file Table_1.docx]

**Supplementary Material**

**Table S1.** Barriers to providing and receiving LGBTQ+ affirming care

| **Domain** | **Subdomain** | **Provider Reported Category** | **Veteran Reported Category** |
| --- | --- | --- | --- |
| Outer Setting | Patient Needs & Resources | Difficult to generalize/assess comfort of other providers | Desire to see/receive more LGBTQ+ affirming care |
|  |  | Providers are not familiar with LGBTQ+ issues | Advocating for own LGBTQ+ affirming care |
|  |  |  | Sexuality does not seem normalized by providers |
|  | External Policies & Incentives | Policy changes with no clinic-level answers |  |
| Inner Setting | Structural Characteristics | Cannot access gender identity information through electronic health record |  |
|  | VA Culture | VA culture of gendered formality | Limited representation |
|  |  |  | Fear of rejection at VA |
|  |  |  | Discrimination from non-LGBTQ+ veterans |
|  |  | Provider habits |  |
|  |  | LGBTQ+ veterans feel they cannot be open and honest | Misgendering by staff/providers |
|  |  |  | Structural discrimination |
|  | Learning Climate | No time to attend trainings/events |  |
|  |  | Need more education |  |
|  | Access to Knowledge | Evolving nature of LGBTQ+ affirming care |  |
|  |  | Providers who need the most information likely are not reached because trainings are often “preaching to the choir” |  |
|  | Available Resources | Limited psychological testing |  |
|  |  | Limited endocrinology |  |
|  |  | Limited fertility clinics |  |
|  |  | Limited support groups |  |
|  |  | Cannot provide gender-affirming surgeries |  |
|  |  | Difficulty with community referrals |  |
|  |  | Contract facility |  |
| Individual Characteristics | Individual LGBTQ+ Affirming Practices | Discomfort related to other veterans who are discriminating against LGBTQ+ veterans |  |
|  | Knowledge & Beliefs | Uncertainty in delivering LGBTQ+ affirming care |  |
|  |  | Lack of experience with LGBTQ+ population |  |
| Health Equity | Culturally Relevant Factors |  | Family is non-affirming |
|  | Clinical Encounter | Concerns about making someone uncomfortable or offending | Negative experience with provider |
|  |  |  | Lack of autonomy from healthcare providers |
|  |  | Worries related to not doing enough for LGBTQ+ veterans | Emotional labor |
|  |  |  | Limited interactions |
|  | Societal Influences | Discrimination from non-LGBTQ+ veterans | Costs of LGBTQ+ affirming care outside of VHA |
|  |  | Harassment/danger in the community | Religion |
|  |  | Military history | COVID-19 |
|  |  | History of LGBTQ+ identity as mental illness | Discrimination outside of the VA |
|  |  | Political climate | Political climate |
|  |  | Socioeconomic status | Historical trauma |
|  |  | Lack of LGBTQ+ representation in the community | Closed mindedness in community towards LGBTQ+ people |
|  |  | Non-affirming providers in the community | Limited representation in rural environments |
|  |  |  | Racial considerations |

**Table S2.** Facilitators to providing and receiving LGBTQ+ affirming care.

| **Domain** | **Subdomain** | **Provider Reported Category** | **Veteran Reported Category** |
| --- | --- | --- | --- |
| Outer Setting | Patient Needs & Resources | Perceived competence of other providers | Knowledge of own needs/health issues as an LGBTQ+ veteran |
|  |  | Providers on my team are comfortable | Receives LGBTQ+ affirming care |
|  |  | Providers are knowledgeable about LGBTQ+ veteran issues | Advocating for own LGBTQ+ affirming care |
|  | External Policies & Incentives | Policy changes led to more LGBTQ+ Veterans accessing VHA care |  |
|  |  | Providers are aware of directives/policies |  |
| Inner Setting | VA Culture | General availability of LGBTQ+ affirming care | Acceptance from providers and staff |
|  |  | VA sends message of inclusivity | Quality healthcare |
|  |  | Availability of inclusive clinic spaces |  |
|  | Learning Climate | Knowledgeable colleague |  |
|  |  | Comfortable speaking out about LGBTQ+ affirming care |  |
|  |  | Communities of practice |  |
|  | Access to Knowledge | Awareness of resources |  |
|  |  | Formalized training |  |
|  |  | Informal training |  |
|  |  | Safety signals |  |
|  | Available Resources | Community referrals for gender-affirming care |  |
| Individual Characteristics | Individual LGBTQ+ Affirming Practices | Open to feedback/correction |  |
|  |  | Asks names and pronouns |  |
|  |  | Tailor to individuals |  |
|  |  | Display safety signals |  |
|  | Knowledge & Beliefs | Always learning |  |
|  |  | Comfort in delivering LGBTQ+ affirming care |  |
| Health Equity | Culturally Relevant Factors |  | Cultural value of “passing” |
|  | Clinical Encounter | Be as compassionate as possible | Positive experience with provider |
|  |  | Apologize and take responsibility |  |
|  | Societal Influences | Free to be themselves | Openness of community |
